# Electrochemical Detection for Uric Acid Based on β-Lactoglobulin-Functionalized Multiwall Carbon Nanotubes Synthesis with PtNPs Nanocomposite

**DOI:** 10.3390/ma12020214

**Published:** 2019-01-10

**Authors:** Bingkai Han, Meixin Pan, Xinran Liu, Jian Liu, Teng Cui, Qiang Chen

**Affiliations:** The Key Laboratory of Bioactive Materials Ministry of Education, College of Life Science, Nankai University, Weijin Road No. 94, Tianjin 300071, China; hanbingkai@mail.nankai.edu.cn (B.H.); 2120171090@mail.nankai.edu.cn (M.P.); 2120181119@mail.nankai.edu.cn (X.L.); 2120181142@mail.nankai.edu.cn (J.L.); 2120181137@mail.nankai.edu.cn (T.C.)

**Keywords:** β-lactoglobulin, multiwall carbon nanotubes, nanocomposite, urate oxidase, uric acid biosensor

## Abstract

In this work, a simple and highly selective electrochemical biosensor for determination of uric acid (UA) is synthesized by using β-lactoglobulin (BLG)-functionalized multiwall carbon nanotubes (MWCNTs) and a platinum nanoparticles (PtNPs) nanocomposite. Urate oxidase (UOx) can oxidize uric acid to hydrogen peroxide and allantoin, which provides a good opportunity for electrochemical detection for UA. Under the optimized conditions, the current changes by the UOx/Bull Serum Albumin (BSA)/BLG-MWCNTs-PtNPs/Glassy Carbon (GC) electrode with the electrochemical method was proportional to the concentration of UA. According to experiments, we obtained a linear response with a concentration range from 0.02 to 0.5 mM and achieved a high sensitivity of 31.131 μA mM^−1^ and a low detection limit (0.8 μΜ). Meanwhile, nanoparticles improved the performance of the biosensor and combined with BLG not only prevented the accumulation of composite nanomaterials, but also provided immobilization of uricase through electrostatic adsorption. This improves the stability and gives the constructed electrode sensing interface superior performance in UA detection.

## 1. Introduction

In the treatment and diagnosis of many diseases, using biosensors to detect uric acid in humans is of vital importance, due to the biosensor’s many advantages, such as low cost, rapid response, high sensitivity, direct detection, and great selectivity. Biosensors have shown their great contribution in flourishing the concept of chemically modified electrodes [1]. The most common way to improve the performance of biosensors is to modify nanocomposite sensing material on the electrode surfaces [2]. Conductive nanomaterials have unique morphologies with high specific surface areas that usually result in very exclusive advantages such as enhanced electronic conductivity and response to sensor applications [3]. Their synthesis and chemical modification offer unlimited possibilities.

Multiwalled carbon nanotubes (MWCNTs) can be thought of as multiple rolled-up graphene nanoribbon sheet structures that generally have diameters of 2–100 nm [4,5]. MWCNTs have been widely used in nanomaterials due to their advantages of mechanical stability, electrical conductivity, and optical properties. In addition, scientific research has found that MWCNTs can contribute to the “mild” enzymatic catalytic pathway of biodegradation [6,7]. Since MWCNTs can serve as a good surface architecture for biosensors, they are widely used by biosensors researchers in fields such as the environment, medicine, bioanalysis, and agriculture [8,9,10,11,12,13]. The chemical modification of MWCNTs and other materials has a direct influence on their physical and chemical properties, which are very important for the electrochemical sensors for biomolecules [14]. A number of studies have been published using MWCNTs-based biosensors to detect different substances and they all showed remarkable results [15,16,17,18,19,20,21,22]. Meanwhile, not satisfied with the use of pure carbon materials, scientists focused on nanocomposite with metal nanoparticles such as Au [23], Ag [24], CuO [25], Pt [26], and Pd [27], so as to further improve their sensitivity, conductivity, and catalytic properties. Among these, platinum nanoparticles (PtNPs) offer many advantages, including good conductivity and a strong ability to catalyze molecules. However, simple chemical modifications of nanomaterials may cause biocompatibility problems in in vivo systems. Therefore, the introduction of biological materials is an inevitable trend for future development. Additionally, the use of proteins to synthesize functional MWCNTs appears to be a sensible choice.

β-Lactoglobulin (BLG) has many biological functions. It is an important globular protein extracted from bovine milk in the lipocalin family of many ruminants and consists of 162 amino acids [28]. There is a surface hydrophobic pocket on its structure so BLG is able to present different hydrophilicity and amphiphilic properties [29]. Our previous study used BLG to adhere to a solid surface and synthesize a BLG-MWCNTs-Gold nanoparticles nanocomposite for glucose detection. The results proved that BLG can improve the dispersibility of hydrophobic particles in solution [30]. Furthermore, as an excellent surfactant with lower toxicity and good biocompatibility, BLG can not only protect the activity of bioactive compounds but can also contribute to the stability of analysis. Common dispersants include poly dimethyl diallyl ammonium chloride and polyvinyl pyrrolidone non-covalently bonded to graphene [31,32].

Uric acid (UA), also known as 2,6,8-trihydroxypurine, is a significant metabolic intermediate in the human body [33]. In a normal state, the concentration of uric acid remains in a stable range, and the quantity of uric acid generated in the body is similar to its metabolic discharge during normal activities. Uric acid is an important marker molecule in serum, urine, and other body fluids, and is used to detect diseases, such as leukemia and pneumonia. The concentration of uric acid is related to purine metabolism [34,35]. Urate oxidase can specifically identify and catalyze uric acid. It contains 34 amino acids and has two T-fold domains with similar structures, which play an important role in the purine metabolism pathway. Different detection areas of uric acid have different concentrations. In serum, normal levels of uric acid are between 2.18 and 7.7 mg/dL. However, in urinary excretion, the concentration will remain within the range 25–74 mg/dL. The concentration of uric acid is an important factor in maintaining long-term stability and upsetting this balance can have serious effects on human cell’s activities. Some diseases, such as gout, high cholesterol, high blood pressure, kidney disease, kidney damage, and cardiovascular disease, are caused by high levels of UA in the blood [36,37,38]. Conversely, other diseases, such as oxidative stress and multiple sclerosis, are caused by abnormally low uric acid levels in humans [39]. Therefore, the detection of uric acid has a vitally important role in human disease diagnosis.

In this work, firstly, based on its amphiphilic property, BLG was combined with MWCNTs so that the MWCNTs would be well distributed. Secondly, modified BLG-MWCNTs that were previously synthesized on the surface of a glassy carbon electrode (GCE) and PtNPs were deposited on the interface by electrodeposition. Finally, we immobilized urate oxidase (UOx) to BLG-MWCNTs-PtNPs/GCE through covalent binding with the help of Bull Serum Albumin (BSA), and used it to study the electrochemical properties of UA. In the present work, we also prepared an innovative UOx/BSA/BLG-MWCNTs-PtNPs/GCE sensing interface for the first time. Based on the results of repeated experiments, we demonstrated a reliable UA sensing system with strong sensitivity, good stability, fast response, simple operation, and a simplified direct detection process that can allow the development and production of a large number of portable uric acid biosensors.

## 2. Materials and Methods

### 2.1. Chemicals and Reagents

Uric acid, urate oxidase, K_2_PtCl_6_, BSA, β-lactoglobulin, dopamine hydrochloride, cysteine (97%), glutathione, urea, and cholesterol were obtained from Sigma Aldrich (Saint Louis, MO, USA). Glucose was obtained from Aladdin Industrial Corporation (Shanghai, China). MWCNTs (450 nm diameter, 10 μm average length, and >95% purity) were obtained from Alpha Nano Technology Co. (Nanjing, China). In this work, all other chemicals were of analytical reagent grade and all relevant experiments used Millipore milli-Q ultrapure water.

### 2.2. Preparation of the UOx/BSA/BLG-MWCNTs-PtNPs/GCE

**Scheme 1:**
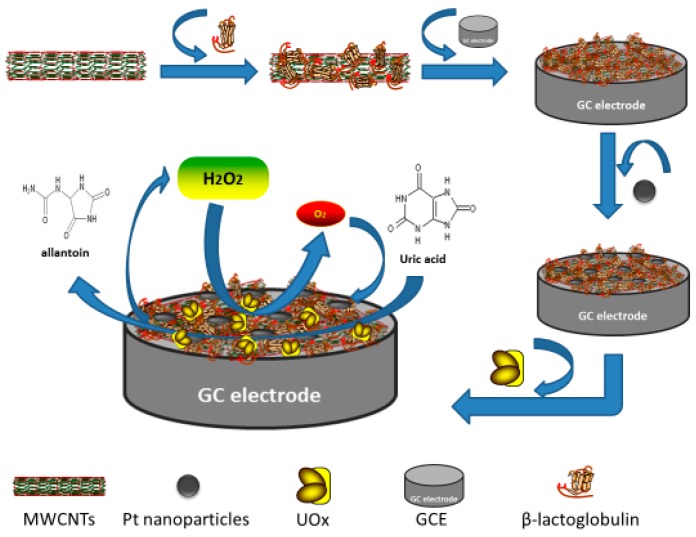
A schematic diagram of the reaction principle and preparation process of the uric acid sensor.

First, MWCNTs were dispersed in BLG solution (2 mg/mL) under ultrasonic agitation for 60 min, so that BLG could functionalize MWCNTs, and the BLG-MWCNTs were obtained by centrifugation at 5000 rpm for 20 min. They were redispersed in ultra-pure water for ultrasonic treatment for subsequent experiments. Then the GC electrode was pre-treated by polishing with 0.3 μm and 50 nm α-alumina powder and ultrasonically cleaned in ethanol and double distilled water for 20 min in order to remove the substance with physical adsorption. The GC electrode was scanned at 0.5 M H_2_SO_4_ under cyclic voltammetry until stable. The electrode was ultrasonically cleaned in doubly distilled water and ethanol for 5 min and blown dry by using N_2_. Next, the GCE was modified with BLG-MWCNTs by adsorption. PtNPs were obtained through electrodeposition (10 mM K_2_PtCl_6_, 0.1 M H_2_SO_4_, −750 mV, 400 s) in order to fabricate BLG-MWCNTs-PtNPs/GCE. Finally, we prepared the UA biosensor by dropping 8 μL UOx (1 mg/mL) mixed with 3 μL BSA (1 mg/mL) onto the BLG-MWCNTs-PtNPs/GCE. The unbound impurities were eluted by Phosphate Buffer Solution (PBS) and stored in a freezer at 4 °C until used in our research. The whole preparation and detection conceptual process is presented in Scheme 1.

### 2.3. Apparatus and Measurements 

The X-ray diffraction (XRD) analysis was obtained using a Rigaku D/max-rA with Cu Kα radiation (λ = 1.5418 Å) (Rigaku, Osaka, Japan). A Tecnai G2 F20 instrument (Philips, Amsterdam, the Netherlands) was used for transmission electron microscopy (TEM) and energy-dispersive X-ray spectroscopy (EDX). We used a Tensor 37 FT-IR (Bruker, Karlsruhe, Germany) to obtain Fourier Transform Infrared Spectroscopy (FT-IR) spectra. A working electrode (GC electrode), counter electrode (Ag/AgCl; saturated KCl), and reference electrode (platinum wire) constituted a conventional three-electrode system. The 283 Potentiostat/Galvanostat electrochemical workstation (EG&G PARC with M270 system, Boston, MA, USA) provided all the electrochemical measurements.

## 3. Results and Discussion

### 3.1. Morphologies of BLG-MWCNTs-PtNPs Nanocomposites

**Figure 1:**
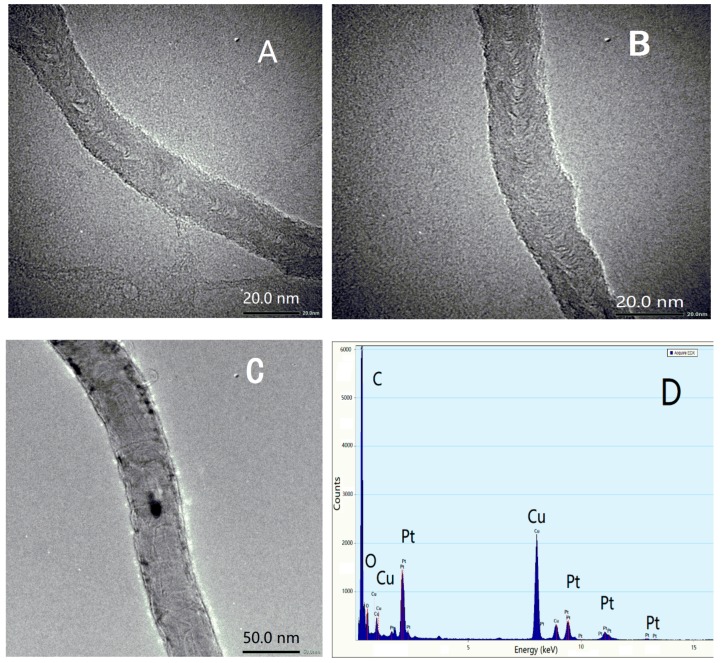

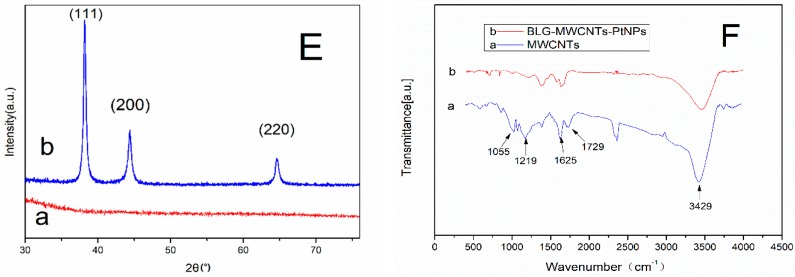
TEM results of MWCNTs (**A**), BLG-MWCNTs (**B**), and BLG-MWCNTs-PtNPs (**C**); EDX spectrum (**D**) of BLG-MWCNTs-PtNPs nanocomposite; XRD result of BLG-MWCNTs-PtNPs (b) and MWCNTs (a) (**E**); FT-IR result of BLG-MWCNTs-PtNPs (b) and MWCNTs (a) (**F**).

In this study, the morphological characterizations of MWCNTs, BLG-MWCNTs, and BLG-MWCNTs-PtNPs were revealed by TEM and are shown in Figure 1. In order to determine the structure of composite materials, TEM examinations were conducted at different levels of magnification. Figure 1 shows the microstructures of MWCNTs (Figure 1A) and BLG-MWCNTs (Figure 1B). It is known that MWCNTs generally consist of several graphite layers of different diameter, like coaxial cables. There are two disulfide bonds and one free sulfhydryl group in the structure of a BLG, which improves the dispersibility of composite materials. Taking advantage of abundant amino acids from BLG-MWCNTs, it is easier for Pt nanoparticles to anchor to active sites. Figure 1C shows the result of the synthetic nanocomposite. PtNPs are dispersed on the surface of the BLG-MWCNTs without accumulation and agglomeration. We also analyzed the EDX spectra of BLG-MWCNTs-PtNPs (Figure 1D). EDX analysis revealed that the nanocomposite consisted of C, O, Cu, and Pt elements. Cu and O peaks were from the substrate. In conclusion the BLG-MWCNTs-PtNPs nanocomposite was synthesized successfully.

XRD results are shown in Figure 1E. Comparing curve a with curve b, the result shows that curve b has the characteristic peak of Pt nanoparticles corresponding to diffraction peaks (111), (200), and (220). There are no other metals in the composite nanomaterials; the diffraction peaks at 2θ values of 39.5, 44.6, and 64.9 correspond to the crystal plane peaks (111), (200), (220), and (311) of platinum metal. This means that the composition of the composite nanomaterials is as expected. The FT-IR result is shown in Figure 1F. Curve a shows the special absorption peak positions of 3429 cm^−1^, 1729 cm^−1^, 1625 cm^−1^, 1219 cm^−1^, and 1055 cm^−1^ corresponding to absorption peak chemical bonds of O–H, C=O, C=C, C–O–C, and C–O. Curve b shows the absorption peaks at 3429 cm^−1^ (O–H), 1219 cm^−1^ (C–O–C), and 1055 cm^−1^ (C–O) decrease and flatten with the combination of BLG. In addition, the absorption peak at 1729 cm^−1^ (C=O) decreased because PtNPs attached to the materials. When PtNPs were modified onto the materials, they affected the ability of the groups to absorb infrared light. Therefore, the BLG-MWCNTs-PtNPs nanocomposite was successfully prepared.

### 3.2. Electrochemical Activity of Nanocomposite Electrodes

**Figure 2:**
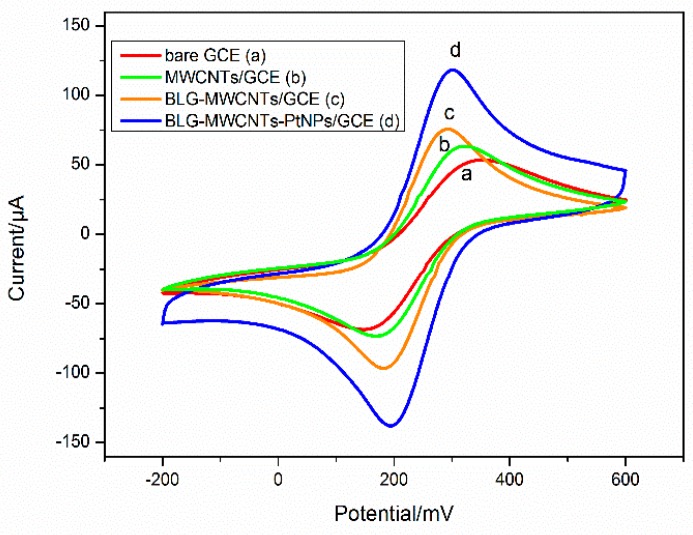
Cyclic voltammograms (CVs) of GC electrode (**a**), MWCNTs modified GC electrode (**b**), BLG-MWCNTs modified GC electrode (**c**) and BLG-MWCNTs-PtNPs modified GC electrode (**d**) scanned in 0.1 M KCl solution with 10 mM [Fe(CN)_6_]^3−^.

Figure 2 shows the cyclic voltammogram (CV) responses of different modified electrodes. Two trends of significant redox peaks can be observed at 190 and 290 mV. We can analyze the electroactive surface area of the modified electrodes by comparing I_p_ based on the Randles-Sevcik equation [40]:

I_p_ = 2.69 × 10^5^AD^1/2^n^3/2^v^1/2^C (1)

Redox peak current, I_p_, can be calculated where A is the electrode’s electroactive surface area, D is the diffusion coefficient (6.70 ± 0.02) × 10^−6^ cm^2^ s^−1^, n is the number of electrons participating equal to 1, v is the scan rate, and C is the concentration of the probe molecule (10 mM) [27]. With reference to the above equation and CVs, the electroactive surface area of the BLG-MWCNTs-PtNPs/GC electrode can be calculated to be 1.6 times higher than that of the BLG-MWCNTs/GC electrode, 1.9 times higher than that of the MWCNTs/GC electrode, and 2.2 times higher than that of the bare GC electrode. These data undeniably show that BLG-MWCNTs-PtNPs/GC electrodes are excellent media for electron transfer between the working electrodes and [Fe(CN)_6_]^3−^. This excellent result was attributed to the synergistic effects of BLG and PtNPs functional MWCNTs, such as large surface area, high electrical conductivity, large edge, basal ration, and catalytic activity.

### 3.3. Optimization of the Testing Environment

**Figure 3:**
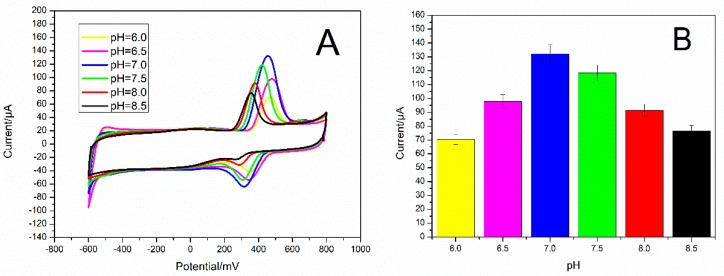
(**A**) CV responses to 0.9 mM uric acid in various pH conditions (pH = 6.0, 6.5, 7.0, 7.5, 8.0, and 8.5) with UOx/BSA/BLG-MWCNTs-PtNPs/GCE (buffer pH = 7.0, the scanning voltage range −600~800 mV), trends are shown in a histogram (**B**).

Figure 3 shows the optimization results of the pH of uric acid detection with the UOx/BSA/BLG-MWCNTs-PtNPs/GCE. The scan rate of uric acid detection is very important. The current response of different scan rates will produce different results. Figure S1 shows the CV results in the same uric acid solution conditions (buffer pH = 7.0, scanning voltage range −600~800 mV, 0.9 mM) under different scan rates. We selected the representative coordinate point scanning rate from 10 to 100 mV/s. The current value is proportionally related to the scanning rate. As the scanning rate increased, the current response also increased. However, if the scan rate is too high, it will affect the modified electrode’s stability, especially the activity of the uric acid oxidase, and shorten the life of the sensor. After a long period of experimentation and contrast, we selected a scanning rate of 50 mV/s as the best scanning rate for the system to detect uric acid.

In order to obtain a more intuitive understanding of the influence of pH change, we also studied different pH environments. Figure 3A shows for various pH gradients (pH = 6.0, 6.5, 7.0, 7.5, 8.0, and 8.5) the response to 0.9 mM uric acid. We can see that within the range of pH = 6.0 to pH = 7.0, the electrodes current response of uric acid increased as the pH value was increased. However, when the pH value was greater than 7.0, the current response of uric acid declined gradually. Figure 3B illustrates that the testing environment of pH = 7.0 is the optimal pH for catalytic activity of uric acid oxidase.

### 3.4. Response of UOx/BSA/BLG-MWCNTs-PtNPs/GCE to Uric Acid

Based on the constructed UOx/BSA/BLG-MWCNTs-PtNPs/GCE, under optimal conditions, we used the cyclic voltammetry method for continuous scanning of the uric acid solution in different concentrations ranging from 0 to 3.0 mM. UA was detected in pH 7.0 phosphate buffer solution and results were shown in Figure S2. Oxidation peaks near 400 mV were observed in the solution containing UA. The current value enhanced gradually with the increase in UA concentration, which indicated that UA was oxidized by the UOx. Voltage optimization results also prove that the optimal operating voltage is 400 mV, which is shown in Figure S3. Therefore, 0.4 V could be used as the working voltage for UA detection.

**Figure 4:**
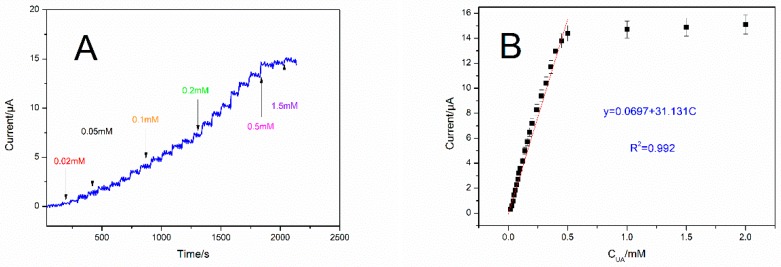
(**A**) Amperometric response after continuous progressive addition of uric acid (UA) with UOx/BSA/BLG-MWCNTs-PtNPs/GCE in 0.1 M PBS solution under stirring; (**B**) the calibration curve of the relationship between UA concentration and amperometric value.

Figure 4A shows the amperometric response after continuous progressive addition of uric acid with UOx/BSA/BLG-MWCNTs-PtNPs/GCE (0.1 M PBS, pH 7.0) at working potential 0.4 V. The UA biosensor could reach 95% of the maximum current within 5 s, indicating that it had a rapid response to uric acid. Through the calibration curve of the UA biosensor displayed in Figure 4B, we see that UA concentration had a linear relationship with current response from 0.02 to 0.5 mM. The result can be summarized as y = 0.0697 + 31.131C (R^2^ = 0.992). In this linear regression, y represents the measured current value and the concentration of UA is indicated as C. Sensitivity is calculated to be 31.131 μA mM^−1^. According to the definition from International Union of Pure and Applied Chemistry (IUPAC), a signal is considered to be credible when the signal-to-noise ratio is equal to 3 [41]. Based on this theory, the detection limit of the UA biosensor was 0.8 μΜ (S/N = 3).

Compared with other studies listed in Table 1, the UOx/BSA/BLG-MWCNTs-PtNPs/GCE electrode system shows superiority in terms of detection limit or linear range and improves the uric acid detection level in the biosensor area.

### 3.5. Reproducibility, Stability, and Selectivity of the UA Biosensor

**Figure 5:**
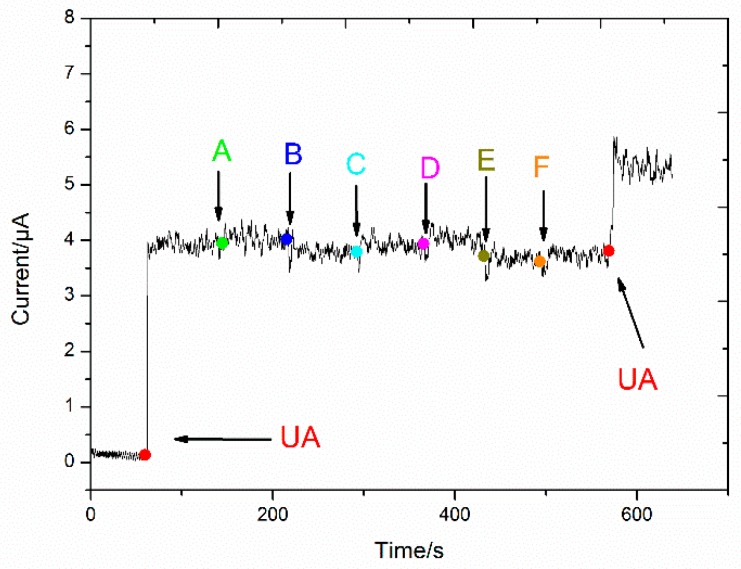
Interference experiments of UOx/BSA/BLG-MWCNTs-PtNPs/GCE in pH 7.0 0.1 M PBS solution containing UA or other substances: (**A**) dopamine (0.1 mM), (**B**) glutathione (0.2 mM), (**C**) cysteine (0.2 mM), (**D**) glucose (0.1 mM), (**E**) urea (0.2 mM), (**F**) cholesterol (0.2 mM).

In order to assess the reproducibility of the UOx/BSA/BLG-MWCNTs-PtNPs/GCE response to UA, we experimented with curve calibration by executing five sequences and achieved results with a relative standard deviation value of 3.8%. The electrostatic adsorption of BLG and UOx provides a good environment for the enzyme, which is why the result showed a good repeatability of the UA biosensor. We also explored the stability of the UOx/BSA/BLG-MWCNTs-PtNPs/GCE system, recording the current value of 0.5 mM UA detection by using the UOx/BSA/BLG-MWCNTs-PtNPs/GCE every three days until the 19th day under the same conditions (stored at 4 °C). The current values are 15.4 µA, 15.2 µA, 14.8 µA, 14.7 µA, 14.3 µA, 14.0 µA, and 13.9 µA, respectively. After 19 days, the current value fell to 90.3% of the first day of detection, showing a relatively stable decline. Finally, we studied the selectivity of the UA biosensor. We investigated some interfering substances that could possibly have influence, such as dopamine, glucose, cysteine, glutathione, urea, and cholesterol. By using the chronoamperometry method, when 0.1 mM uric acid was adding, the current responded rapidly and remained stable. Interfering substances were then added one by one to assess the effect on the biosensor response. Figure 5 shows the comparison; UA caused a significant current decrease according to the UOx/BSA/BLG-MWCNTs-PtNPs/GCE. Therefore, the results demonstrated that interference of these substances can be neglected, meaning that they had no obvious interference effect on UA detection, and the developed method provided excellent selectivity and reliability for the UA biosensor.

### 3.6. Analysis of Uric Acid in Biological Samples

An experiment was designed to investigate the application of the UA biosensor to biological samples. The modified Pt electrode interface was used to test in human serum (20-fold diluted with buffer). Normally, the uric acid level in human’s serum is in the range of 0.13–0.46 mM. If the concentration of uric acid in the body exceeds this level, it can cause complications. The experiments were conducted using the standard addition method and the results of three samples are shown in Table 2.

This demonstrated that the data provided by the UA biosensor with the UOx/BSA/BLG-MWCNTs-PtNPs/GCE modified interface were excellent, with recovery ranging from 95.5% to 107.33%. The RSD was also in an excellent range of 2.97–4.18%. All these results demonstrate the promising application potential of our biosensor.

## 4. Conclusions

In conclusion, we developed a highly selective and simple electrochemical biosensor for the determination of uric acid based on UOx catalysis with a UOx/BSA/BLG-MWCNTs-PtNPs/GCE for the first time. Based on a UOx/BSA/BLG-MWCNTs-PtNPs/GCE sensing interface, the sensor achieved a good linear relationship between the electrochemical current response and concentration of uric acid. The innovative use of BLG not only improved performance but also presented great potential for biosensors, especially for detection in vivo. We proposed an innovative method for real-time dynamic multi-index detection by integration, with excellent biocompatibility, materials that could play a vitally important role in both medical and scientific research. Meanwhile, this study provided a powerful new method for the electrochemical detection of uric acid and also made a great contribution to broadening application prospects for the development of UA biosensors in the future.

## Figures and Tables

**Table 1:** Characteristics of various amperometric uric acid biosensors.

Immobilization Materials	Detection Technique	Detection Limit (µM)	Linear Range (µM)	pH	Reference
Platinum–nickel/multiwalled carbon nanotubes	Cyclic voltammetry	0.03	0.1–240.4	7.0	[42]
Polyethylenimine-multiwall carbon nanotubes/GCE	Differential pulse voltammetry	0.1	0.5–50	7.4	[43]
Graphene oxide-uricase/GCE	Cyclic voltammetry	3.45	20–490	6.5	[44]
Graphite screen-printed electrodes/Prussian blue/poly(4-aminosalicylic acid)/uricase	Chronoamperometry	3.0	10–200	8.27	[45]
Cellulose acetate deposited screen-printed carbon electrode pre-modified with cobalt phthalocyanine	Chronoamperometry	15	15–250	9.2	[46]
Screen-printed electrode modified with Prussian blue/uricase	Chronoamperometry	10	30–300	8.5	[36]
UOx/BSA/BLG-MWCNTs-PtNPs/GCE	Chronoamperometry	0.8	20–500	7.0	This work

**Table 2:** Recovery ratios of uric acid in 20% serum.

Sample	Added UA (mM)	Calculated UA (mM)	Recovery (%) UA	RSD (%) UA
1	0.200	0.191	95.50	2.97
2	0.300	0.322	107.33	4.18
3	0.400	0.423	105.75	3.45

RSD: Relative Standard Deviation.

## Supplementary Materials

The following are available online at http://www.mdpi.com/1996-1944/12/2/214/s1, Figure S1: CVs of the scan rate of UA detection with UOx/BSA/BLG-MWCNTs-PtNPs/GCE in the same uric acid solution conditions (buffer pH = 7.0, the scanning voltage range −600~800 mV, 0.9 mM) under different scan rates (10 mV/s, 20 mV/s, 30 mV/s, 40 mV/s, 50 mV/s, 60 mV/s, 80 mV/s, 100 mV/s); Figure S2: CVs study of UOx/BSA/BLG-MWCNTs-PtNPs/GCE upon different concentrations of UA (0, 0.15, 0.3, 0.45, 0.6, 0.9, 1.2, 1.5, 1.8, 2.1, 3.0 mM) into pH 7.0, 0.1 M PBS solution under stirring; Figure S3: Amperometric response of UOx/BSA/BLG-MWCNTs-PtNPs/GCE upon successive additions of 50 µM uric acid into 0.1 M PBS solution under stirring at operating voltages from 250 mV to 450 mV; the contrast of sensitivity is shown in a bar graph (B).
