# Supplementary material for: Electrochemical Detection for Uric Acid Based on β-Lactoglobulin-Functionalized Multiwall Carbon Nanotubes Synthesis with PtNPs Nanocomposite
Source: Materials (Basel). 2019 Jan 10;12(2):214. doi: 10.3390/ma12020214 (PMC6356623; doi:10.3390/ma12020214)
Supplement: Supplementary file 1 [file materials-12-00214-s001.pdf]

Supplementary Materials: Electrochemical Detection for Uric Acid Based on β-Lactoglobulin-Functionalized Multiwall Carbon Nanotubes Synthesis with PtNPs Nanocomposite

Bingkai Han, Meixin Pan, Xinran Liu, Jian Liu, Teng Cui and Qiang Chen *

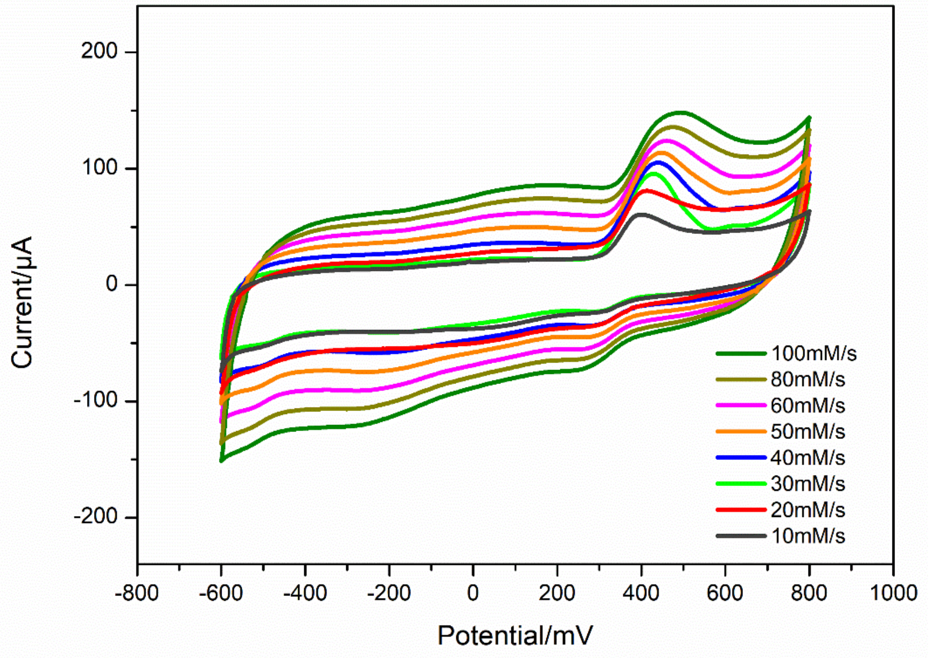

Figure S1. CVs of the scan rate of UA detection with UOx/BSA/BLG-MWCNTs-PtNPs/GCE in same uric acid solution conditions (buffer pH = 7.0, the scanning voltage range −600 mV~800 mV, 0.9 mM) under different scan rate (10 mV/s, 20 mV/s, 30 mV/s, 40 mV/s, 50 mV/s, 60 mV/s, 80 mV/s, 100 mV/s).

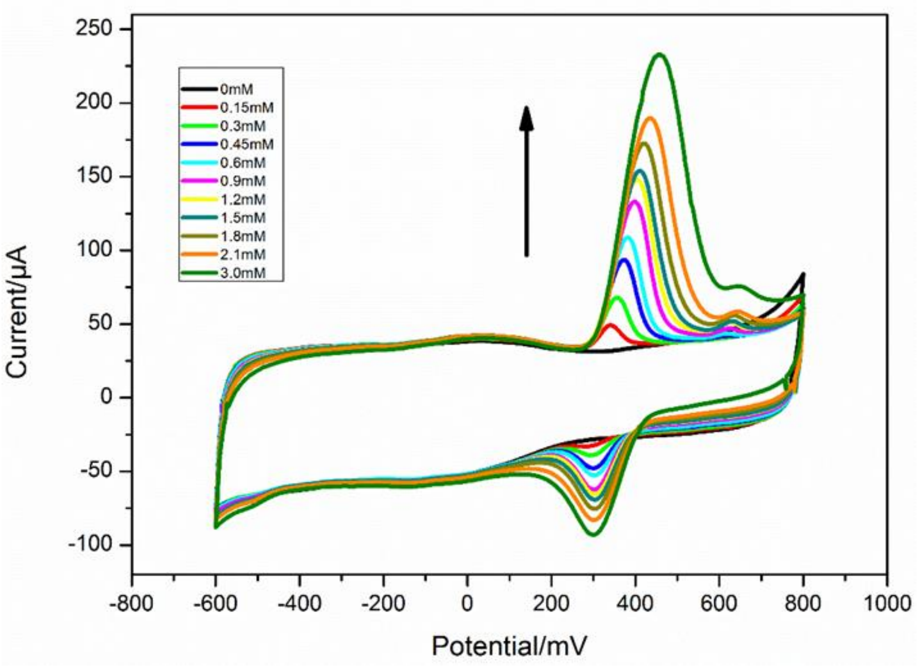

Figure S2. CVs study of UOx/BSA/BLG-MWCNTs-PtNPs/GCE upon different concentrations of UA (0, 0.15, 0.3, 0.45, 0.6, 0.9, 1.2, 1.5, 1.8, 2.1, 3.0 mM) into pH 7.0, 0.1 M PBS solution under stirring

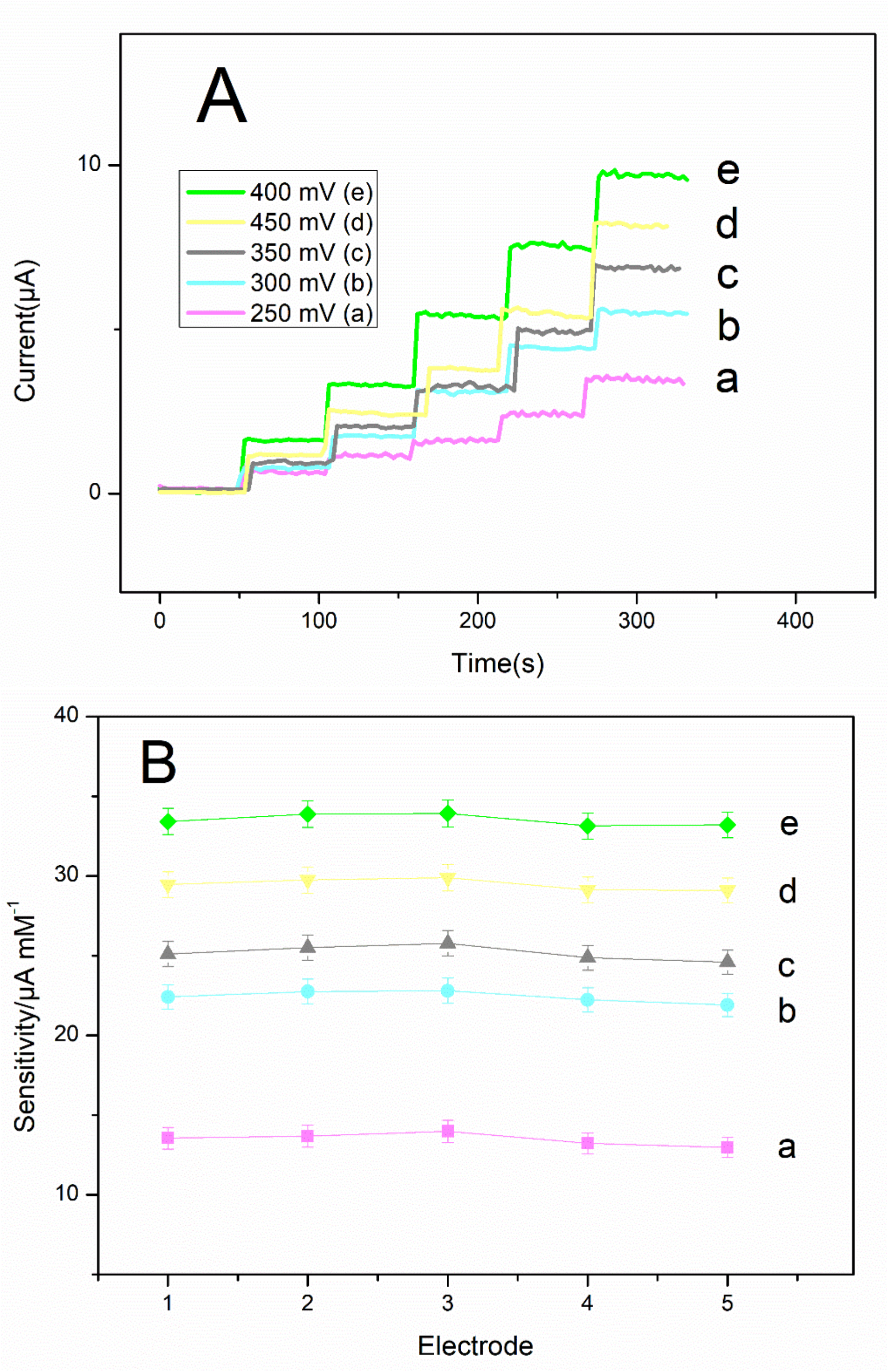

Figure S3. (**A**) Amperometric response of UOx/BSA/BLG-MWCNTs-PtNPs/GCE upon successive additions of 50 μM uric acid into 0.1 M PBS solution under stirring at operating voltage from 250 mV to 450 mV, the contrast of sensitivity is shown in a bar graph (**B**).
